# CRANet: a comprehensive residual attention network for intracranial aneurysm image classification

**DOI:** 10.1186/s12859-022-04872-y

**Published:** 2022-08-05

**Authors:** Yawu Zhao, Shudong Wang, Yande Ren, Yulin Zhang

**Affiliations:** 1 College of Computer Science and Technology, China University of Petroleum, Qingdao, Shandong China; 2 The Department of Medical Imaging Center, The Affiliated Hospital of Qingdao University, Qingdao, Shandong China; 3 College of Mathematics and System Science, Shandong University of Science and Technology, Qingdao, Shandong China

**Keywords:** Medical image classification, Deep residual learning, Comprehensive attention mechanism

## Abstract

Rupture of intracranial aneurysm is the first cause of subarachnoid hemorrhage, second only to cerebral thrombosis and hypertensive cerebral hemorrhage, and the mortality rate is very high. MRI technology plays an irreplaceable role in the early detection and diagnosis of intracranial aneurysms and supports evaluating the size and structure of aneurysms. The increase in many aneurysm images, may be a massive workload for the doctors, which is likely to produce a wrong diagnosis. Therefore, we proposed a simple and effective comprehensive residual attention network (CRANet) to improve the accuracy of aneurysm detection, using a residual network to extract the features of an aneurysm. Many experiments have shown that the proposed CRANet model could detect aneurysms effectively. In addition, on the test set, the accuracy and recall rates reached 97.81% and 94%, which significantly improved the detection rate of aneurysms.

## Introduction

The analysis and processing of medical images play an essential role in preventing and diagnosing diseases. Improving the classification performance of medical images is a common problem in medical research. Intracranial aneurysms (IAs) are cerebral blood vessels' pathological expansion. Its incidence is second only to cerebral thrombosis and hypertensive cerebral hemorrhage. About 3% of healthy people suffer from intracranial aneurysms [1]. Standard inspection methods for IAs include digital subtracted angiography (DSA), computed tomography angiography (CTA), and magnetic resonance angiography (MRA). In recent years, advances in medical imaging technology have significantly increased the chance of finding an aneurysm. However, because the boundary between the tumor and normal tissue is not clear, accurately identifying the location of an aneurysm is a knowledge-intensive task, and training a radiologist for tumor diagnosis can be costly and time-consuming. In recent years, deep learning has shown superiority in various computer vision tasks, including medical image analysis [2, 3] and object classification [4, 5]. Therefore, deep learning models with automatic recognition and classification can assist imaging experts in analyzing aneurysm images more effectively.

With the development of deep learning technology, convolutional neural networks emerge endlessly, from LeNet5 [6] to AlexNet [7], and then from VGGNet [8] to GoogleNet [9], and later ResNet [10]. The emergence of these models enables convolutional neural networks to make breakthroughs in solving image classification problems. For example, Ker et al. [11] proposed an automatic classification of histological sections of the brain and breast using Google Inception V3 convolutional neural network, which improves classification performance. Literature [12] applied 3D CNN to brain scan classification of CT and MRI images and achieved good results. CNN is a practical feature extraction method with an impressive performance in various application fields [13, 14].

However, it is worth noting that the continuous deepening of the network may cause the gradient to disappear and explode during the backpropagation process, which diverges the network and affects the test results. To solve the above problems, the ResNet network came into being. The shallow information is introduced into the deep layer to ensure image information transmission by introducing the residual structure. Wang et al. [15] suggested a new metastatic cancer image classification method based on the ResNet model, which could effectively alleviate the problems of gradient explosion and gradient disappearance, therefore, significantly improving the performance of cancer diagnosis. Roy et al. [16] proposed an improved residual network based on attention, which captured the spatial features of spectral images in an end-to-end training method and used an effective feature calibration mechanism to improve classification performance. Liu et al. [17] proposed a deeply integrated network with an attention mechanism, which significantly improved the success rate of early diagnosis of glaucoma. Jafar et al. [18] constructed a hyperparameter-based approach based on ResNet and CNN structures and showed that it resulted in significant performance improvements. Qiao et al. [19] proposed a simple and effective residual learning intelligent diagnosis system for diagnosing whether the fetus had congenital heart disease. Ghaderzadeh et al. [20]. suggested a model based on a deep convolutional neural network (CNN) to distinguish acute lymphoblastic leukemia (ALL) from benign causes and achieved good classification results. Although these residual models have achieved good results, they still have some problems, such as low computational efficiency, low accuracy, and unreliable diagnosis.

Generally speaking, deep convolutional neural networks can learn deeper features of medical images and are also robust. However, most deep learning methods mainly learn features from the entire image, including tumor information, background information, and noise. This makes the extracted features poorly different and inaccurate model classification and wastes computing resources. To solve these problems, Vaswani [21] proposed a new simple network architecture, which connects the encoder and decoder in the complex recurrent neural network through the attention mechanism, and achieved great success. The attention mechanism has shown many advantages in computer vision [22–25]. It does not process the information of the entire image at once but learns certain areas of the image through the attention module and extracts more essential features. Huang et al. [26] used cross-attention and channel attention to enhance the interdependence of features in spatial and channel dimensions, respectively and improved the quality of the generated image. Some works use visual feature maps and convolution kernels to understand the decision-making of CNN in image classification tasks [27–29]. The SE module using channel attention is not only applied to image classification but also to semantic segmentation [30, 31] and image title [32], and other fields. Roy et al. [33] introduced spatial and channel attention mechanisms to enhance meaningful features. Based on the above methods, one or two attention mechanisms are mainly added to improve the model's performance, and the impact of the position of multiple attention mechanisms in the network on the classification performance is not considered. To solve this problem, this paper studies the influence of the positional relationship of the attention mechanism in the deep residual network structure on the classification results.

Aiming at the 2-classification (T: without aneurysm and F: with aneurysm) and 4-classification (L: aneurysm diameter greater than 7 mm, S: aneurysm diameter less than 3 mm, M: aneurysm diameter between 3-7 mm, and T: without aneurysm) problems of MRA aneurysm images, the main contributions of this paper are as follows:

Firstly, a deep residual neural network with a comprehensive attention mechanism is proposed, called CRANet. The contribution of each area of the image to the network is different, so a spatial attention mechanism is added to give extra attention to each area. In the process of training the network, not every feature map is helpful to the improvement of classification performance. A channel attention mechanism is added to obtain the importance of each feature map. Combining the two attention mechanisms with the ResNet model is used to improve the performance of aneurysm classification.

Secondly, the ResNet network is improved. In different positions of the network, two attention mechanisms are added. As the depth of the network increases, the feature information will be lost to different degrees. The attention mechanism can control the features in the network to improve classification performance.

Finally, many experiment results show that the model's results are better than the structure that does not use the attention mechanism or only uses part of the attention mechanism.

## Data and methods

### Datasets and preprocessing

The data set used in this article contains MRA 3-D images of 678 patients from 9 different devices. The size of the 3-D images of each patient is different, and their size varies between 448 × 448 and 696 × 768. Moreover, each 3-D image contains 128 to 152 different 2-D slices. Among the 678 patients, there are 578 patients with aneurysms and 100 regular patients without an aneurysm. Among patients with aneurysms, they can be roughly divided into three categories according to the diameter of the aneurysm, S category: the aneurysm diameter is less than 3 mm, M category: the aneurysm diameter is between 3 and 7 mm, L category: the aneurysm diameter is greater than 7 mm.

**Fig. 1:**
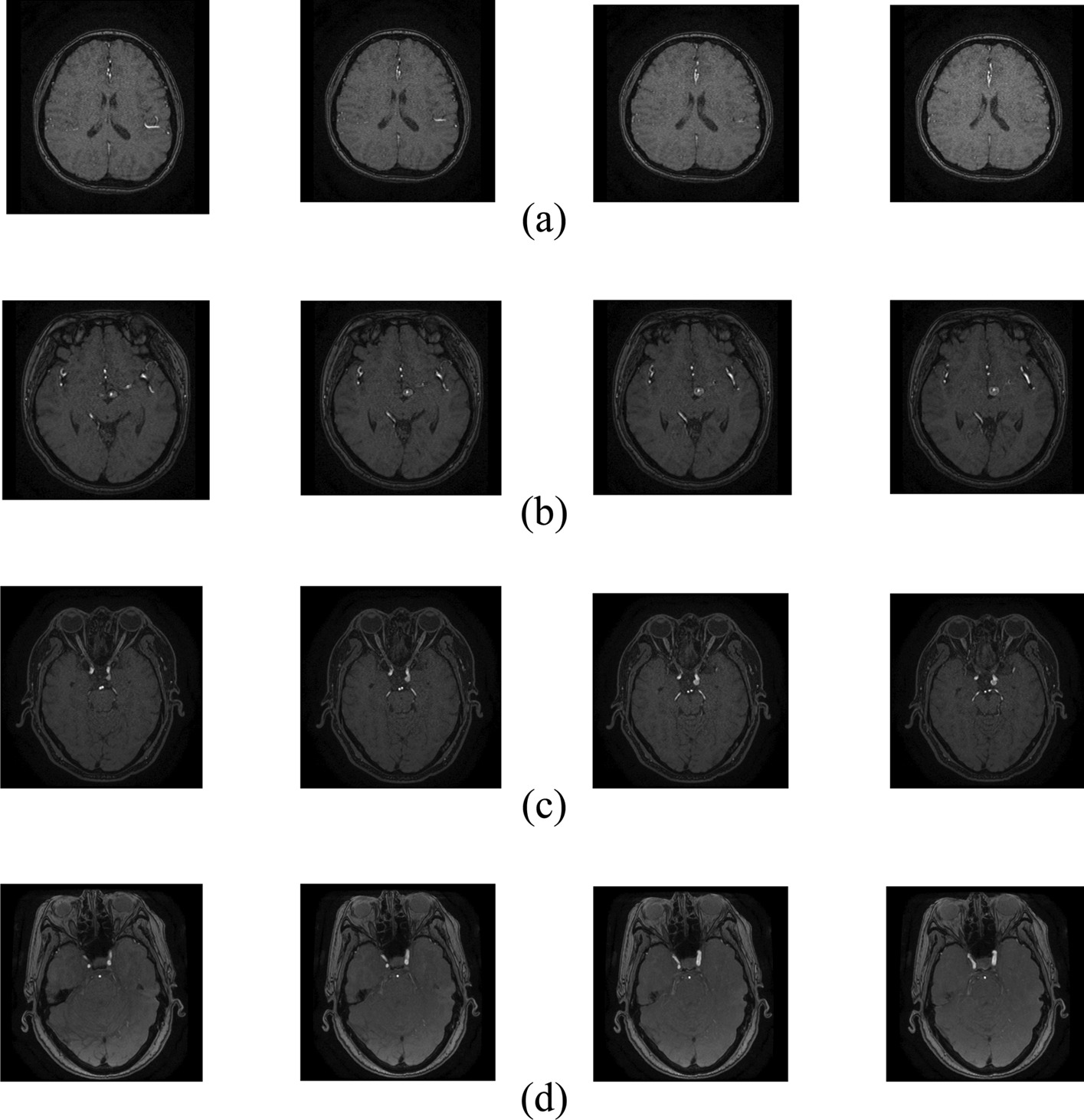
Image slice of 2-D MRA aneurysm. (**a**) represents an aneurysm image of an average person; (**b**) represents type L, the aneurysm diameter is greater than 7 mm; (**c**) represents type M, the aneurysm diameter is between 3 and 7 mm; and (**d**) represents type S, the aneurysm diameter is less than 3 mm

This paper randomly selects 10,000 two-dimensional image slices and divides them into a training set, a validation set, and a test set, according to the ratio of 8:1:1, to improve the model's generalization ability. The image size is randomly cropped to 260 × 260 and standardized, using random horizontal flipping, vertical flipping, angle rotation (between −30 degrees and 30 degrees), and adjusting brightness, contrast, and saturation method enhance the training data. The slice image of each type of MRA aneurysm is shown in Fig. 1.

### Model structure

**Fig. 2:**
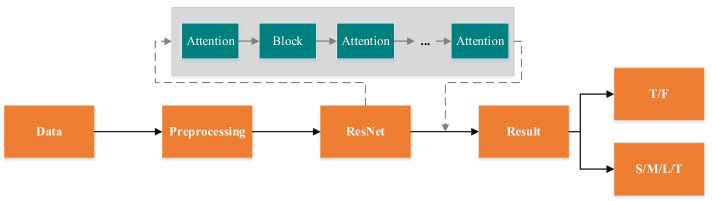
The overall experimental structure diagram

**Fig. 3:**
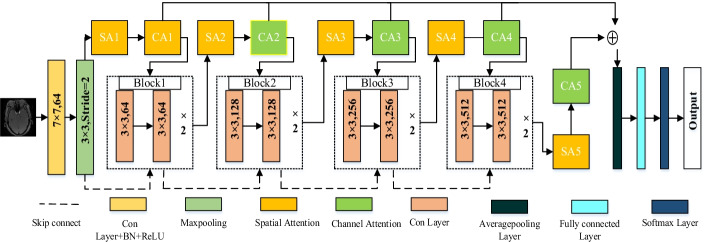
Structure diagram of mixed attention model

**Fig. 4:**
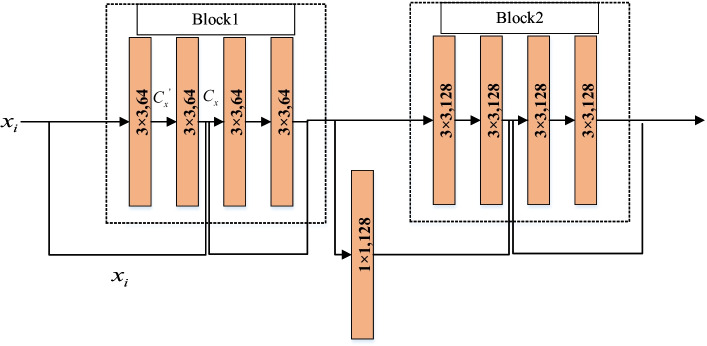
Residual network structure

Medical image processing is highly valued by researchers worldwide. Medical image classification is an essential research direction in medical image processing, since. It is the condition for reasonable evaluation and appropriate treatment plans for patients, and it is gradually playing an increasingly important role in the medical field. Deep learning reduces the requirement for manual extraction of features. MRA aneurysm images first use image enhancement and other techniques to increase their features. Then through the mixed attention ResNet network, they can learn helpful information autonomously so that the entire system can output the best Classification results. The experimental flow structure is shown in Fig. 2, where ''Attention'' represents the combination of residual spatial attention and residual channel attention. ''Block'' means there are multiple convolutional layers per Block. More details are shown in Figs. 3 and 4.

The attention mechanism has been applied to different scenarios to solve different problems in computer vision. Zhao et al. [34] advanced a two-stage segmentation network of global and local attention modules and fully convolutional networks to solve the uneven distribution of image boundaries. Oktayet [35] and others combined the spatial attention model with the U-Net network for pancreas separation. In previous studies, only the attention mechanism was added. However, it did not pay attention to the effect of the attention mechanism's position in the network model on the classification performance. Therefore, this paper will integrate the attention mechanism applied to different network locations to observe their performance.

**Fig. 5:**
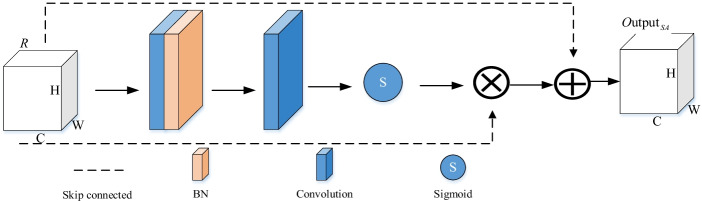
Residual spatial attention model

**Fig. 6:**
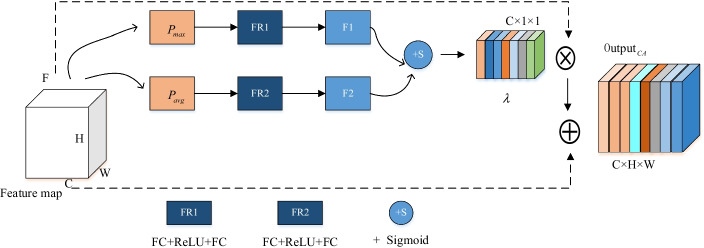
Residual channel attention model

All experiments in this article are based on the ResNet network model, as shown in Fig. 3. Our network structure includes five-channels attention modules, five spatial attention modules, and four Block blocks. The channel attention module (CA1-5) can weigh the feature maps output by the upper layer to obtain more essential channel features. The spatial attention module (SA1-5) can enhance attention to a specific area on the feature map while suppressing background information and outside areas. The Block is an integral part of the ResNet network model. Each Block includes four convolutional layers and uses a 3 × 3 convolution kernel. There is a 1 × 1 convolutional layer between every two-Block. In addition, the residual connection structure is added to ensure that the image information will not be lost during the convolution operation. The softmax function can display the results of multi-classification in the form of probability to realize the classification of aneurysms. More details of these different modules are shown in Figs. 4, 5, and 6.

In the process of information transmission, deep neural networks may cause problems such as loss of feature information, the disappearance of gradients, gradient explosion, and network non-convergence. By introducing residual structure, these problems can be alleviated to a large extent. The deep residual network can directly pass the parameters to the subsequent layers over the middle layers, reducing the network's complexity, solving the deep network's degradation problem, and improving network performance. The residual network comprises a convolutional layer, batch normalization (BN), and the essential parts of an activation function. The convolutional layer is used for feature extraction, and the convolution kernel is set to downsample the information to reduce the amount of calculation. During the training process, the data distribution will change with the update of the training parameters, so the BN operation can be used to adaptively adjust the network parameters to solve the impact of data offset or increase. The activation function adds more nonlinearity and can fit more complex tasks. Figure 4 shows the details of the residual structure.

Compared with the network model that does not adopt the skip structure, the residual network of this structure can retain the characteristic information to the greatest extent. If the $x_{i}$ dimension is different from the learning residual function dimension, a 1 × 1 convolution kernel can increase or decrease the dimension. Then, the output of the residual structure can be defined as:

1 $$C_{x} = C_{x}^{^{\prime}} + x_{i}$$

where, $x_{i}$ represents the input feature map, $C^{^{\prime}}_{x}$ represents the residual term, and $C_{x}$ represents the optimal mapping.

#### Residual spatial attention model

The residual spatial attention model can make the regions with similar characteristics enhance each other, to highlight the tumor area in the global field of vision. The specific details are shown in Fig. 5. A coefficient of [0, 1] can be obtained through the sigmoid function, through which different weights are assigned to each channel or space so that the network can assign different degrees of importance to each feature map.

We let $R^{C \times H \times W}$ be the input of the spatial attention module, C is the number of feature maps, and H and W are expressed as height and width, respectively. The input features are processed through convolution and BN operations, and the spatial attention coefficient S is obtained using the Sigmoid function. The formula is expressed as follows:

2 $$S{ = }s{\text{igmoid}}(C{\text{onv}}^{7 \times 7} R^{C \times H \times W} )$$

In this paper, the input feature map and the obtained spatial attention feature map are fused through the residual structure to obtain the final spatial attention feature map.

3 $$O{\text{utput}}_{SA} { = }S \cdot R + R$$

#### Residual channel attention model

We introduced a channel attention model to find the best feature channel, which can strengthen the attention to related channels while suppressing irrelevant channel feature maps. The specific details are shown in Fig. 6.

We let F represent the input of the channel attention model. Then, we use the maximum pooling and average pooling layers to obtain global information and compress the H × W two-dimensional matrix into n number, representing the characteristics of the matrix. FR1 consists of two fully connected layers and a ReLU layer. The channel size of the first fully connected layer is C/r(r = 2), and the channel size of the second fully connected layer is C. There is a ReLU layer between the two fully connected layers. FR2 and FR1 have the same structure. The obtained feature maps F1 and F2 are added together, and the channel attention coefficient $\lambda$ is obtained through the Sigmoid function.

4 $$\lambda { = }sigmoid[FR1(P_{avg} ) + FR2(P_{max} )]$$

Then multiply the input feature map by the channel attention coefficient $\lambda$ to give each channel a different weight, and add it to the input feature map to obtain the final channel attention map $Output_{CA}$.

5 $$Output_{CA} = \lambda \cdot F + F$$

### Evaluation methods

To better evaluate the classifier's performance, three Accuracy, recall, and F1 standards are used to assess the model's classification performance [19]. The F1 score combines precision and recall, a comprehensive assessment of CRANet, so the higher the F1the better CRANet's classification performance. The larger the recall value, the higher the sensitivity to the tumor. The F1 score ranges from 0 to 1, where 1 represents that CRANet has the best performance. The calculation formula of the Accuracy is as follows:

6 $${\text{Accuracy = }}\frac{TP + TN}{{P + N}}$$

where: TP is the number of correctly classified malignant samples, TN is the number of correctly classified benign samples, P is the number of malignant samples, and N is the number of benign samples.

We predicted not only the overall accuracy rate, but also the accuracy rate of each category. The calculation formula is as follows:

7 $$ACC_{i} = \frac{{S_{i} }}{S}$$

where:$ACC_{i}$ represents the prediction accuracy rate of each category, $S_{i}$ represents the number of correct predictions for each category, and $S$ represents the total number.

The recall represents the proportion of judging malignancy as malignant, and the recall calculation formula is as follows:

8 $$recall = \frac{TP}{{TP + FN}}$$

where: FN is the number of samples that are malignant and misclassified as benign.

F1 is used to judge the valid benign rate, and the F1 calculation formula is as follows:

9 $$F1 = 2 \cdot \frac{precision \cdot recall}{{precision + recall}}$$

where: $precision = TP/TP + FP$. FP represents the number of tumor images judged to be non-tumor images.

## Experimental results

In this paper, the experimental environment is as follows: all models in this article use the PyTorch deep learning framework to train the model. The GPU is NVIDIA Tesla V100, and the video memory is 32 GB. The model hyperparameters are set as follows: the Adam algorithm is used to optimize the loss function. The deep learning model is trained with small-batch samples. The batch_size is set to 32. A fixed-step strategy is used to adjust the learning rate during the training process. The initial learning rate is set to 0.0001. The gamma value is 0.85. In addition, L2 regularization is added to impose penalty constraints on the weight parameters. The penalty coefficient is set to 0.0001. Then, the parameters are determined by setting different epochs.

**Fig.7:**
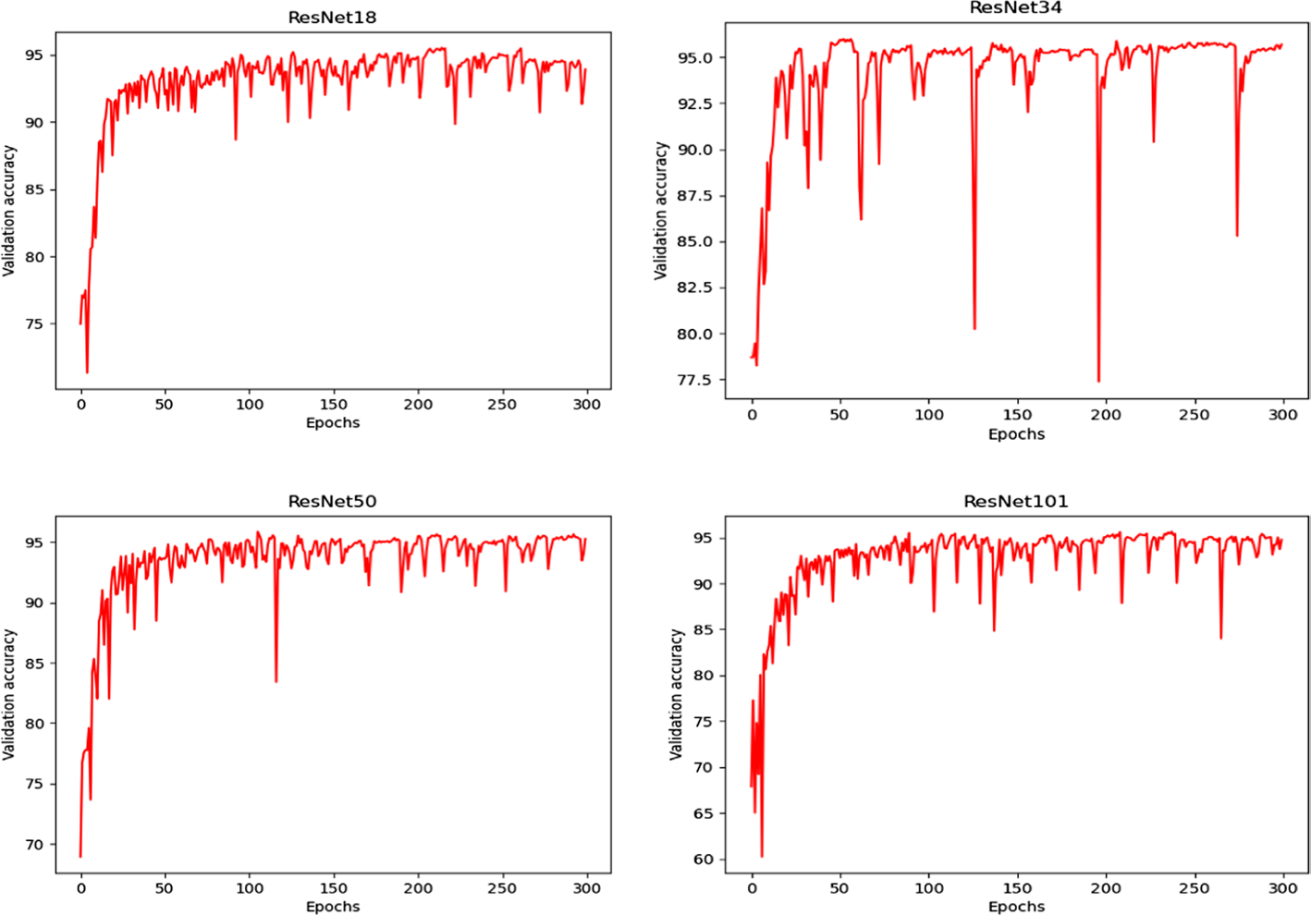
The accuracy curve of the model

This paper performs experiments on the ResNet18, ResNet34, ResNet50, and ResNet101 network models. The optimal classification performance is obtained by setting different Epochs, and the optimal model is added to the attention model at different positions for further analysis. The experimental results are shown in Table 1. To obtain a reliable and stable network model, this article experiments on the verification set to verify the generalization ability and the accuracy of the model. Each experiment was repeated five times to reduce the error of the experiment and ensure the reliability of the experimental results. Then the average was taken as the final result. The experimental results are shown in Fig. 7.

**Table 1:** The influence of the number of iterations of each model on the Accuracy (%)

Epoch	ResNet18	ResNet34	ResNet50	ResNet101
50	94.18 ± 1.3	95.55 ± 2.8	94.44 ± 0.6	94.63 ± 0.7
100	95.85 ± 1.6	95.88 ± 1.6	95.57 ± 0.8	94.43 ± 0.3
150	96.20 ± 1.8	95.18 ± 1.2	96.39 ± 0.6	95.26 ± 0.7
200	96.57 ± 0.9	96.10 ± 0.2	95.64 ± 0.13	95.23 ± 0.5
250	96.51 ± 0.5	95.62 ± 0.3	95.91 ± 0.4	95.16 ± 0.3
300	94.18 ± 0.9	95.87 ± 0.2	95.65 ± 0.2	95.49 ± 0.1

It can be seen from Table 1 that the ResNet18 network model performs the best, achieving an accuracy of 96.57% when the Epoch is 200. Among them, 96.57% refers to the final experimental result obtained by averaging five experiments, and 0.9 refers to the variance.

It can be seen from Table 1 and Fig. 7 that ResNet18 obtained the best classification effect among the four models, which further proves that the complexity of the model matches the MRA aneurysm image data. Therefore, this article will further study adding different attention models to different positions in the ResNet18 network model on classification performance. To verify that the spatial attention model can effectively improve the classification results, we first add a spatial attention model behind the pooling layer and then add a spatial attention mechanism behind each Block in turn.

We first validate the effect of the spatial attention model on aneurysm classification and add the spatial attention model after the pooling layer and each ''Block'' to observe the change in accuracy. We use SA1, SA1-2, SA1-3, SA1-4, and SA1-5 to represent the different positions of the spatial attention model in the network. For example, the spatial attention model added after the pooling layer is denoted by SA1, and the spatial attention model added after "Block1" is denoted by SA1-2. The experimental results are shown in Table 2.

**Table 2:** The influence of the position and number of spatial attention models on Accuracy (%)

Mode structure	Accuracy
SA1	96.76 ± 0.3
SA1-2	96.66 ± 0.5
SA1-3	96.99 ± 0.2
SA1-4	96.49 ± 0.6
SA1-5	97.21 ± 0.1

Table 2 shows the influence of the spatial attention model on the classification results. At the same time, we also studied the influence of the channel attention model on the model separately. In this comparison, we only introduce the channel attention model and add the channel attention model at different network positions to observe the accuracy change. We use CA1, CA1-2, CA1-3, CA1-4, and CA1-5 to represent the different positions of the channel attention model in the network. For example, the channel attention model added after the pooling layer is denoted as CA1, and the channel attention model added after "Block1" is denoted as CA1-2. The experimental results are shown in Table 3.

**Table 3:** The influence of the position and number of channel attention models on Accuracy (%)

Mode structure	Accuracy
CA1	96.43 ± 0.4
CA1-2	96.55 ± 0.1
CA1-3	96.84 ± 0.4
CA1-4	97.12 ± 0.6
CA1-5	97.36 ± 0.4

It can be seen from Tables 2 and 3 that the space and channel attention models are added to the ResNet18 network model, and the effect has been improved to a certain extent. We combine the two to improve the network structure and call it a comprehensive residual attention network.

In this comparison, we combine two attention models to validate their performance. In Table 4, SA1 + CA1 (CA1 + SA1) means adding spatial attention model and channel attention model (channel attention model and spatial attention model) after the pooling layer, SA1-2 + CA1-2 (CA1-2 + SA1—2) indicates that the spatial attention model and the channel attention model (channel attention model and spatial attention model) are added after both the pooling layer and ''Block1''. The experimental results are shown in Table 4.

**Table 4:** The effect of comprehensive attention model on Accuracy (%)

Mode structure	Accuracy
SA1 + CA1	96.91 ± 0.1
SA1-2 + CA1-2	97.20 ± 0.2
SA1-3 + CA1-3	97.36 ± 0.1
SA1-4 + CA1-4	97.55 ± 0.3
SA1-5 + CA1-5	97.81 ± 0.2
CA1 + SA1	96.64 ± 0.4
CA1-2 + SA1-2	97.11 ± 0.1
CA1-3 + SA1-3	97.26 ± 0.3
CA1-4 + SA1-4	97.42 ± 0.4
CA1-5 + SA1-5	97.63 ± 0.1

**Fig. 8:**
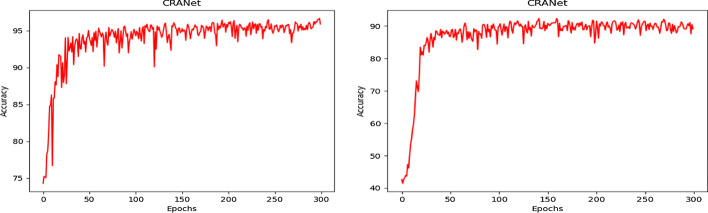
The accuracy change curve of the CRANet model (the left picture is 2-classification, the right picture is 4-classification)

We validate the impact of ResNet18, ResNet34, ResNet50, ResNet101, and CRANet network models on aneurysm classification performance. It can be seen from Table 4 that the improved ResNet18 network using spatial and channel attention has achieved better classification performance. We also used multiple evaluation indicators to evaluate network performance, and the experimental results are shown in Table 5 and Fig. 8.

**Table 5:** Comparison with the residual network models (%)

*ResNet18* [9]									
	ACC	recall	F1	Accuracy		ACC	recall	F1	Accuracy
L	0.89	0.94	0.92		T	0.93	0.99	0.96	
M	0.87	0.91	0.87		F	0.96	0.83	0.89	
S	0.84	0.85	0.86						
T	0.93	0.81	0.97						
Average	0.88	0.87	0.88	91.75	Average	0.94	0.91	0.92	96.57
*ResNet34*									
	ACC	recall	F1	Accuracy		ACC	recall	F1	Accuracy
L	0.82	0.86	0.84		T	0.84	0.96	0.89	
M	1.00	0.86	0.92		F	0.79	0.44	0.56	
S	0.88	0.91	0.89						
T	0.81	0.89	0.85						
Average	0.87	0.88	0.87	90.25	Average	0.81	0.70	0.73	96.10
*ResNet50*									
	ACC	recall	F1	Accuracy		ACC	recall	F1	Accuracy
L	1.00	0.76	0.86		T	0.92	0.96	0.94	
M	0.96	0.79	0.86		F	0.86	0.72	0.78	
S	0.76	1.00	0.86						
T	0.84	0.84	0.84						
Average	0.89	0.85	0.85	89.15	Average	0.89	0.84	0.86	95.64
*ResNet101*									
	ACC	recall	F1	Accuracy		ACC	recall	F1	Accuracy
L	0.89	0.95	0.91		T	0.99	0.82	0.95	
M	0.95	0.68	0.79		F	0.80	0.96	0.87	
S	0.82	0.84	0.83						
T	0.75	0.95	0.84						
Average	0.85	0.85	0.84	89.60	Average	0.89	0.89	0.91	95.23
*CRANet*									
	ACC	recall	F1	Accuracy		ACC	recall	F1	Accuracy
L	0.90	0.90	0.90		T	0.97	0.96	0.97	
M	0.89	0.86	0.87		F	0.88	0.92	0.90	
S	0.91	0.94	0.92						
T	0.95	0.95	0.95						
Average	0.91	0.91	0.91	92.55	Average	0.93	0.94	0.94	97.81

Through a large number of experimental results, we can see that our proposed CRANet has a good effect on the detection of intracranial aneurysms. By using residual spatial attention models, different degrees of attention can be given to different spatial locations of each feature map. Similarly, residual channel attention models can give different degrees of attention to different feature maps. This allows better extraction of tumor features in the feature map, thereby improving the classification performance of aneurysm images. We have conducted experimental verifications on different network models to observe the inaccuracy changes. The detailed experimental results are shown in Table 5. The accuracy and recall rates of 97.81% and 94% were achieved in the two classification problems. The accuracy and recall rates of tumors were 92.55% and 91% for the multi-class classification.

To further verify the validity and rationality of the model, the classical models CNN [5], VGG [8], GoogleNet [9], ResNet [10], InceptionV3 [36], DenseNet [37] are compared with the CRANet model proposed in this paper. The detailed data in Table 6 show that our proposed CRANet network has achieved the best performance in aneurysm classification tasks. ResNet18 performs better than other ResNet variants, reaching 96.57% accuracy, 0.91 recall rate, and 0.92 F1 scores in 2-classification tasks. The 4-classification tasks achieved a 91.75% accuracy rate, 0.87 recall rate, and 0.88 F1 scores. Among VGG, Googlenet, InceptionV3, and DenseNet models, the Inception model can extract higher-level semantic features and enrich feature information. Thus it performs best in 2-classification and 4-classification tasks. The accuracy rate, recall rate, and F1 score were 97.16%, 0.91, and 0.92 in the 2-classification task, and 91.68%, 0.87, and 0.86 in the 4-classification. In the CNN model, 95.89% accuracy rate, 0.87 recall rate, and 0.85 F1 scores were obtained in the 2-classification task, and 86.27% accuracy rate, 0.82 recall rate, and 0.81 F1 scores were obtained in the 4-classification. It can be seen that spatial and channel attention can well focus on the regions with significant features, indicating that our method is an effective strategy for classification tasks.

**Table 6:** Performance comparison of each model

Models	2-classification			4-classification		
	Accuracy	Recall	F1	Accuracy	Recall	F1
ResNet18	96.57	0.91	0.92	91.75	0.87	0.88
ResNet34	96.10	0.70	0.73	90.25	0.88	0.87
ResNet50	95.64	0.84	0.86	89.15	0.85	0.85
ResNet101	95.23	0.89	0.91	89.60	0.85	0.84
VGG	96.64	0.89	0.88	90.85	0.85	0.86
GoogleNet	96.87	0.89	0.89	86.50	0.81	0.79
InceptionV3	97.16	0.91	0.92	91.68	0.87	0.86
DenseNet	96.32	0.89	0.87	88.79	0.84	0.84
CNN	95.89	0.87	0.85	86.27	0.82	0.81
CRANet (Our model)	97.81	0.94	0.94	92.55	0.91	0.91

As shown in Table 6, on the aneurysm two classification and four classification problems, the proposed model improves the aneurysm classification performance. Compared with some classical models, the model proposed in this paper improves the detection performance of aneurysms to a certain extent. It has been proved by a large number of experiments that the model is very effective for the classification of aneurysms.

## Conclusion and future work

This paper proposed a mixed residual attention learning model for intracranial aneurysm detection called CRANet. In addition, using the residual structure in the convolutional neural network can effectively avoid the loss of information and make the detection error smaller. The CRANet model can identify whether the image is an aneurysm image and distinguish the type of aneurysm better. CRANet to better assist doctors in diagnosing aneurysms, reduce doctors' workload, and improve doctors' work efficiency.

In future work, we will collect more aneurysm images and use a deeper network structure to learn deeper features to improve the robustness and accuracy of the model. Then an automatic segmentation network model will be designed to diagnose the structure and location of the aneurysm. In addition, this will have a positive effect on clinical practice. At the same time, it will be of great significance to human health.

## Data Availability

The data used in this paper are from the People's Hospital of Huangdao District, Qingdao, and the aneurysm images are collected from nine different devices. The datasets generated during and analyzed during the current study are not publicly available due to we have signed a non-disclosure agreement with the hospital to protect patient information, but are available from the corresponding author on reasonable request.

## References

[1] Vlak MH, Algra A, Brandenburg R (2011). Prevalence of unruptured intracranial aneurysms, with emphasis on sex, age, comorbidity, country, and time period: a systematic review and meta-analysis. Lancet Neurol.

[2] Singh SP, Wang L, Gupta S (2020). 3D deep learning on medical images: a review. Sensors.

[3] Ker J, Wang L, Rao J (2017). Deep learning applications in medical image analysis. Ieee Access.

[4] Yadav SS, Jadhav SM (2019). Deep convolutional neural network based medical image classification for disease diagnosis. J Big Data.

[5] Badža MM, Barjaktarović MČ (2020). Classification of brain tumors from MRI images using a convolutional neural network. Appl Sci.

[6] LeCun Y, Bottou L, Bengio Y (1998). Gradient-based learning applied to document recognition. Proc IEEE.

[7] Krizhevsky A, Sutskever I, Hinton GE (2012). Imagenet classification with deep convolutional neural networks. Adv Neural Inf Process Syst.

[8] Simonyan Karen, Zisserman Andrew. Very deep convolutional networks for large-scale image recognition. 2014, arXiv preprint arXiv:1409.1556.

[9] Szegedy C, Liu W, Jia Y et al. Going deeper with convolutions[C]//Proceedings of the IEEE conference on computer vision and pattern recognition. 2015;1–9.

[10] He K, Zhang X, Ren S, et al. Deep residual learning for image recognition[C]//Proceedings of the IEEE conference on computer vision and pattern recognition. 2016;770–8.

[11] Ker J, Bai Y, Lee HY (2019). Automated brain histology classification using machine learning. J Clin Neurosci.

[12] Ker J, Singh SP, Bai Y (2019). Image thresholding improves 3-dimensional convolutional neural network diagnosis of different acute brain hemorrhages on computed tomography scans. Sensors.

[13] Dhillon A, Verma GK (2020). Convolutional neural network: a review of models, methodologies and applications to object detection. Progr Artif Intell.

[14] Singh SP, Wang L, Gupta S (2020). Shallow 3D CNN for detecting acute brain hemorrhage from medical imaging sensors. IEEE Sens J.

[15] Wang M, Gong X. Metastatic cancer image binary classification based on resnet model[C]//2020 IEEE 20th international conference on communication technology (ICCT). IEEE, 2020;1356–9.

[16] Roy SK, Manna S, Song T, Bruzzone L (2021). Attention-based adaptive spectral-spatial Kernel resnet for hyperspectral image classification. IEEE Trans Geosci Remote Sens.

[17] Liu Y, Yip LWL, Zheng Y (2022). Glaucoma screening using an attention-guided stereo ensemble network. Methods.

[18] Jafar A, Myungho L. Hyperparameter optimization for deep residual learning in image classification[C]//2020 IEEE international conference on autonomic computing and self-organizing systems companion (ACSOS-C). IEEE, 2020;24–9.

[19] Qiao S, Pang S, Luo G (2022). RLDS: an explainable residual learning diagnosis system for fetal congenital heart disease. Futur Gener Comput Syst.

[20] Ghaderzadeh, Mustafa, et al. A fast and efficient CNN model for B‐ALL diagnosis and its subtypes classification using peripheral blood smear images. Int J Intell Syst. 2022;37.8:5113–33.

[21] Vaswani A, Shazeer N, Parmar N, et al. Attention is all you need. Adv Neural Inform Process Syst. 2017. p. 5998–6008.

[22] Wang F, Jiang M, Qian C et al. Residual attention network for image classification[C]//Proceedings of the IEEE conference on computer vision and pattern recognition. 2017;3156–64.

[23] Woo S, Park J, Lee JY et al. Cbam: Convolutional block attention module[C]//Proceedings of the European conference on computer vision (ECCV). 2018;3–19.

[24] Ciompi F, de Hoop B, van Riel SJ (2015). Automatic classification of pulmonary peri-fissural nodules in computed tomography using an ensemble of 2D views and a convolutional neural network out-of-the-box. Med Image Anal.

[25] Hu J, Shen L, Sun G. Squeeze-and-excitation networks[C]//Proceedings of the IEEE conference on computer vision and pattern recognition. 2018;7132–41.

[26] Huang Z (2020). CaGAN: a cycle-consistent generative adversarial network with attention for low-dose CT imaging. IEEE Trans Comput Imag.

[27] Chen R, Chen H, Ren J et al. Explaining neural networks semantically and quantitatively[C]//Proceedings of the IEEE/CVF international conference on computer vision. 2019;9187–96.

[28] Zhou B, Khosla A, Lapedriza A, et al. Learning deep features for discriminative localization[C]//Proceedings of the IEEE conference on computer vision and pattern recognition. 2016;2921–29.

[29] Springenberg JT, Dosovitskiy A, Brox T et al. Striving for simplicity: the all convolutional net[J]. arXiv preprint arXiv:1412.6806, 2014.

[30] Li K, Wu Z, Peng KC et al. Tell me where to look: guided attention inference network[C]//Proceedings of the IEEE conference on computer vision and pattern recognition. 2018;9215–23.

[31] Fu J, Liu J, Tian H etal. Dual attention network for scene segmentation[C]//Proceedings of the IEEE/CVF conference on computer vision and pattern recognition. 2019;3146–54.

[32] Lu J, Xiong C, Parikh D et al. Knowing when to look: adaptive attention via a visual sentinel for image captioning[C]//Proceedings of the IEEE conference on computer vision and pattern recognition. 2017;375–83.

[33] Roy AG, Navab N, Wachinger C. Concurrent spatial and channel ‘squeeze & excitation’ in fully convolutional networks[C]//International conference on medical image computing and computer-assisted intervention. Springer, Cham, 2018;421–9.

[34] Zhao Y, Li P, Gao C (2020). TSASNet: tooth segmentation on dental panoramic X-ray images by two-stage attention segmentation network. Knowl-Based Syst.

[35] Oktay O, Schlemper J, Folgoc LL et al. Attention u-net: learning where to look for the pancreas. arXiv preprint arXiv:1804.03999, 2018.

[36] Szegedy C, Vanhoucke V, Ioffe S et al. Rethinking the inception architecture for computer vision[C]//Proceedings of the IEEE conference on computer vision and pattern recognition. 2016;2818–26.

[37] Huang G, Liu Z, Van Der Maaten L et al. Densely connected convolutional networks[C]//Proceedings of the IEEE conference on computer vision and pattern recognition. 2017;4700–8.

